# Qualitative Evaluation of Divergent Thinking in Patients with Schizophrenia

**DOI:** 10.1155/2005/386932

**Published:** 2006-02-27

**Authors:** Takahiro Nemoto, Masafumi Mizuno, Haruo Kashima

**Affiliations:** Department of NeuropsychiatrySchool of MedicineKeio UniversityTokyoJapan

## Abstract

Patients with schizophrenia show deficits across a broad spectrum of neurocognitive domains. In particular, deficits in verbal fluency are common. Verbal fluency tests are neuropsychological tests that assess frontal lobe function or executive function but also assess divergent thinking. However, few studies have considered the impairment of verbal fluency from the viewpoint of divergent thinking. To consider the structure of divergent thinking, not only verbal assessments but also non-verbal assessments are indispensable. We administered several fluency tests, the idea fluency test, the design fluency test, and word (letter and category) fluency tests to 26 patients with schizophrenia and 26 healthy control subjects to evaluate divergent thinking in both groups and assessed their responses qualitatively. An acceptable minimal level of intelligence was maintained in the patient group. Although attention and executive functioning were relatively preserved in the subjects with schizophrenia, they demonstrated significant deficits in divergent thinking and had particular difficulty in producing ideas and designs requiring concept flexibility, a conversion of viewpoint, originality, or novelty. Research on deficits in divergent thinking in patients with schizophrenia may contribute to the development of cognitive and behavioral rehabilitation programs.

## 1. Introduction

Cognitive deficits are important features of schizophrenia because they fundamentally relate to the psychiatric symptoms and functional outcomes [1,2,17,18]. Patients with schizophrenia show deficits across a broad spectrum of neurocognitive domains [22,44]. In particular, deficits in verbal fluency are common [3,5,10, 19,28], and such deficits are said to reflect essential and characteristic symptoms of schizophrenia, like poverty of speech, flattened affect, anhedonia, and so on [32, 35,38,45].

Fluency tests measure one's ability to generate various products. Divergent thinking is a way of thinking proposed by Guilford [21] that is used to answer questions without fixed answers, in contrast to convergent thinking used to search for the sole right answer. Therefore, Guilford [20] mentioned fluency tests that consisted of open-ended questions as one type of representative tasks to evaluate divergent thinking. Guilford [21] advocated the structure of an intellect model in an attempt to organize all human intellect along three dimensions: "operations", which can be performed as thought process; "contents", to which operations can be applied; and "products", which might result from performing operations on different content categories. He regarded divergent thinking as an "operation". Guilford also described the association between divergent thinking and creativity and extracted six factors from creative thinking: fluency, flexibility, originality, elaboration, sensitivity to problems, and redefinition [20].

However, few studies have considered the impairment of fluency in patients with schizophrenia from the viewpoint of divergent thinking. Further research on the divergent thinking deficits in schizophrenia may reveal the nature of impairment and may provide concrete clues to strategies for cognitive remediation for their impairments. Few studies on fluency in patients with schizophrenia have used fluency tests that consist of other modalities besides words. To further examine the divergent thinking deficits, non-verbal assessments are also indispensable. Although the design fluency test by Jones-Gottman and Milner [25] is a well-known non-

ISSN 0953-4180/05/$17.00 © 2005 – IOS Press and the authors. All rights reserved verbal fluency test, this test has been criticized for its overly vague and global scoring procedure [42]. Therefore, we modified the method of scoring the design fluency test.

In this study, we administered a fluency test battery consisting of several fluency tests (the idea fluency test, the design fluency test, and word fluency tests) to evaluate divergent thinking in patients with schizophrenia and evaluated the patients' responses to each test using the original criteria described for each test. We have already reported the research using word and idea fluency tests [50]. The subjects in the previous study were older, and they had longer disease duration than those in the current study. In the present study with younger subjects, we used three kinds of fluency tests and aimed to evaluate the impairment of divergent thinking in patients with schizophrenia extensively and in detail.

We hypothesized that qualitative evaluation of responses in each fluency test could reveal impairment of divergent thinking in patients with schizophrenia. Moreover, we intended to reveal that divergent thinking abilities are relatively independent of other neuropsychological abilities.

## 2. Subjects and methods

### *2.1. Subjects*

Twenty-six Japanese outpatients with schizophrenia (17 men, 9 women) and 26 healthy control subjects (19 men, 7 women) were recruited for the current study (Table 1). Healthy control subjects were volunteers. The patients were diagnosed by two trained psychiatrists using the ICD-10 criteria [49]. Patients had a mean age of 29.6 years (SD = 5.9) and a mean formal education of 13.9 years (SD = 1.8). They had been ill for a mean period of 59.9 months (SD = 49.8), and the mean age of onset was 24.7 years old (SD = 6.4). All patients were taking neuroleptics, and the mean chlorpromazine-equivalent dose was 474.0 mg/day (SD = 273.1). The mean score on the Brief Psychiatric Rating Scale (BPRS) [39] to evaluate psychiatric symptoms was 35.9 (SD = 8.7). No significant differences in gender, age, or education were observed between the two groups.

All subjects were right-handed, according to the Edinburgh Inventory [37]. Subjects with a history of alcoholism, drug abuse, or serious neurological illness were excluded. The patients had no extra-pyramidal symptoms as shown by the score of the Drug Induced Extra-Pyramidal Symptoms Scale (DIEPSS) [24]. None of the healthy control subjects reported any previous psychiatric disorders or familial history of psychosis. The institutional review board approved this study's protocol. After a complete description of the study to the subjects, written informed consent was obtained from each subject.

Table 1
Subjects

| | Schizophrenia | Control |
|---|---|---|
| $N$ (Male/Female) | 26 (17/9) | 26 (19/7) |
| Age | 29.6 (5.9) | 29.7 (5.2) |
| Education period (years) | 13.9 (1.8) | 14.1 (1.5) |
| Age of onset | 24.7 (6.4) | |
| Disease duration (months) | 59.9 (49.8) | |
| Medication dose[a] | 474.0 (273.1) | |
| DIEPSS[b] | 2.7 (2.7) | |
| BPRS[c] | 35.9 (8.7) | |

[a]chlorpromazine-equivalent mg.
[b]Drug Induced Extra-Pyramidal Symptoms Scale.
[c]Brief Psychiatric Rating Scale.

### *2.2. Methods*

Three kinds of fluency tests were administered to evaluate divergent thinking. We developed and defined the response criteria for qualitative evaluations using the idea fluency and design fluency tests. In the fluency tests, the examiner asked each examinee to produce as many responses as possible within a limited time period.

#### *2.2.1. Idea fluency test*

In the idea fluency test, the examiner asked the subject to think of as many uses for empty tin cans as possible in 5 minutes [43,46,50]. Repetitions or irrelevant responses were excluded from evaluation. As shown in Table 2, the responses were classified into three groups: task-dependent responses, task-modified responses, and task-independent responses. We partly modified the criteria of the idea fluency test used in our previous study [50]. Task-dependent responses were regarded as stereotyped ideas immediately suggested by a stimulus object, such as its intended or common uses prepossessed with a parochial viewpoint that tin cans are essentially containers. For example, responses like "an ashtray" or "a vase" were regarded as task-dependent responses. Task-modified responses were regarded as ideas generated by considering the object's shape, such as viewing the empty tin can as a cavity or a cylindrical form, in order to create new uses other than its conventional implementation. This type of response

Table 2
Classification of idea fluency test responses

| | |
|---|---|
| Task-dependent response | Stereotyped ideas immediately suggested by a stimulus object, such as its intended or common uses prepossessed with a parochial viewpoint that tin cans are essentially containers.<br>E.g., "an ashtray", "a vase", "a pan", "an inkstand". |
| Task-modified response | Ideas generated by considering the object's shape, such as viewing the empty tin can as a cavity or a cylindrical form, in order to create new uses other than its conventional implementation.<br>E.g., "a roller", "a lampshade", "a cap", "a wheel". |
| Task-independent response | Ideas that are perceived from the partial characteristics of tin cans, such as their material, and that ignore the receptacle characteristics of cans, such as being a cavity or cylindrical form.<br>E.g., "an ornament", "a mirror", "a paperweight", "a knife". |

requires a conversion of viewpoint and flexibility of thinking, for example, using the tin cans as a roller or a lampshade. Task-independent responses were regarded as ideas that are perceived from the partial characteristics of tin cans, such as their material, and that ignore the receptacle characteristics of cans, such as being a cavity or cylindrical form; for example, responses like "an ornament" or "a mirror" were included in this category. This type of response is derived from transcendent conceptions that rise above the characteristics of the stimulus. Consequently, task-modified and task-independent responses are creative responses and are regarded as "high-quality" responses. Two raters were trained to administer the scorings of the idea fluency test to a high level of reliability [intra-class correlation coefficient (ICC) = 0.979 for the task-dependent responses, ICC = 0.955 for the task-modified responses and ICC = 0.974 for the task-independent responses] for 10 schizophrenia patients and 10 healthy controls.

#### *2.2.2. Design fluency test*

Before administering the design fluency test, the examiner gave the subject white paper and requested the subject to generate as many different drawings that were as meaningless as possible in a 1-minute period as a trial. The subject was asked not to generate namable drawings (e.g., pictures of concrete objects) or merely scribbles. Several examples of acceptable and unacceptable drawings were shown. If the subject did not understand the directions, the examiners repeated them. After confirming the subject's understanding, the examiner gave the subjects sheets of paper printed with 20 sets of four dots placed in a square on each paper (Fig. 1). The subjects were asked to generate as many different drawings as possible that used the four dots. The basis of the scoring system was qualitatively the same as that of the idea fluency test; that is, the responses were classified into three groups: task-dependent responses, task-modified responses, and task-independent responses (Table 3, Fig. 1). Task-dependent responses were regarded as designs immediately suggested by the four dots printed in a square on the paper. Task-modified responses were regarded as designs generated by considering the shape of the four dots as corners of a square. This type of response requires a change in viewpoint and flexibility of design. Task-independent responses were regarded as designs generated from partial characteristics of the dots and ignored the characteristics of the four dots as the corners of a square form. The two raters were trained to administer the scorings of the design fluency test to a high level of reliability [intra-class correlation coefficient (ICC) = 0.999 for the task-dependent responses, ICC = 0.998 for the task-modified responses and ICC = 0.999 for the task-independent responses] for 10 schizophrenia patients and 10 healthy controls.

Table 3
Classification of design fluency test responses

| |
|---|
| A three-step evaluation method was used.<br>1) Evaluation of task-dependent responses<br>Drawings consisting of only a few straight lines connecting the four dots or a few straight lines whose ends located on the square formed by connecting the four dots or that consisted of three straight lines contained by the square formed by the four dots were regarded as drawings that assumed the four dots formed a square. |
| 2) Evaluation of task-independent responses<br>Drawings in which three of the four dots were not located on the edge of the design or at the intersections or ends of lines were regarded as drawings that did not assume the four dots formed a square. |
| 3) Evaluation of task-modified responses<br>Drawings that did not conform to the criteria for the task-dependent and task-independent responses shown above were placed in this category. |
| Appendix<br>Repeated/similar responses, namable responses, and scribbling were not counted. |

#### *2.2.3. Word (Letter and category) fluency tests*

We administered two sorts of word fluency tests, the letter fluency test and the category fluency test. In the letter fluency test, the examiner asked the subjects to

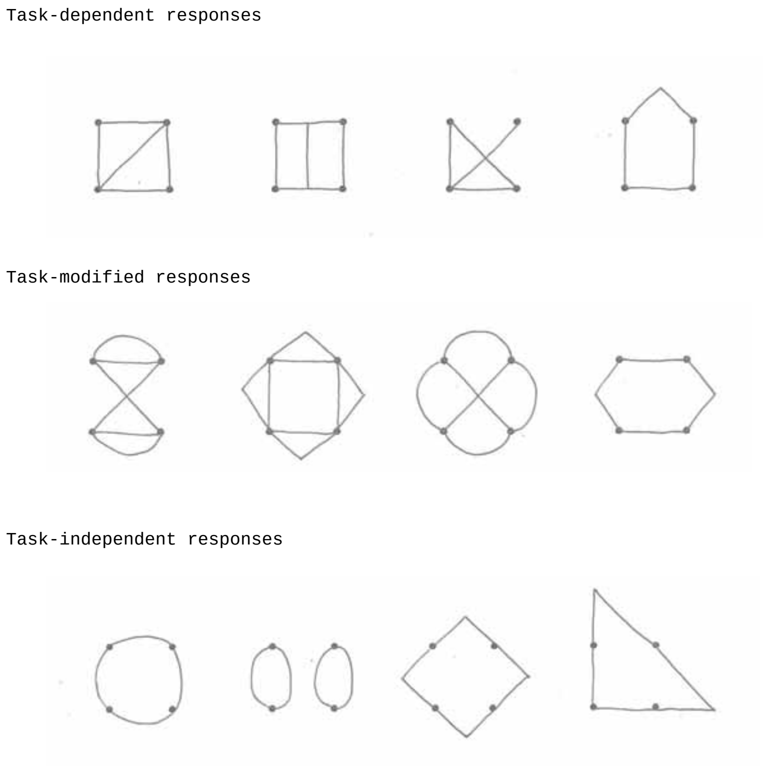

Fig. 1. Examples of design fluency test responses.

say as many words in 60 seconds as they could think of that begin with a given kana (Japanese syllable), excluding proper nouns and numbers. Instead of "F", "A", and "S" [4], we used three kana, "shi", "i", and "re", that have different frequencies of occurrence in the Japanese language. In the category fluency test, animal, fruit, and vehicle naming tasks were each performed within 60 seconds. The reliability of the letter and category fluency tests in their Japanese versions has been evaluated elsewhere [26]. We used the total number of responses to the three tasks as the score for each fluency test.

*2.2.4. Other neuropsychological assessments*

In addition to the fluency tests, several neuropsychological assessments, including the Mini-Mental State Examination (MMSE, total score) [13], the Letter Cancellation Test (LCT, number of correct responses) [11], the Digit Span test (DST, span length) [48], the Rey Auditory Verbal Learning Test (RAVLT, score on the delayed recall trial) [30], and the Wisconsin Card Sorting Test (WCST, number of categories achieved and perseverative errors) [23,27], were administered.

### *2.3. Statistical analyses*

All statistical analyses were carried out using the Statistical Package for the Social Sciences (SPSS) version 12.0J for Windows. The multivariate analysis of variance (MANOVA) was conducted to compare the demographic and cognitive variables between the two groups. Pearson or Spearman correlations were used to evaluate correlations between fluency test scores and neuropsychological/demographic variables, for theoretically-motivated associations.

## 3. Results

### *3.1. Group differences in fluency test scores and other neuropsychological variables*

The results of MANOVA analysis are summarized in Table 4. The analysis revealed a significant overall difference between the healthy control group and the patient group (Wilks' lambda $= 0.34$, F $= 3.92$, df $= 17$, 34, $p < 0.001$). In the idea fluency test, the schizophrenic patients had a significantly lower num-

Table 4
Means and standard deviations of fluency test scores in schizophrenia and control groups

| | Schizophrenia ($n = 26$) | Control ($n = 26$) |
|---|---|---|
| Idea Fluency Test | | |
| Total responses | 10.5 (4.7)[b] | 14.3 (5.5) |
| Task-dependent responses | 5.9 (3.4) | 6.5 (3.2) |
| Task-modified responses | 2.9 (2.2)[b] | 4.3 (2.2) |
| Task-independent responses | 1.7 (1.9)[a] | 3.6 (2.4) |
| Design Fluency Test | | |
| Total responses | 20.5 (8.9) | 22.3 (8.7) |
| Task-dependent responses | 9.9 (7.6)[b] | 5.6 (6.9) |
| Task-modified responses | 9.2 (8.2)[b] | 14.1 (8.5) |
| Task-independent responses | 1.4 (1.9) | 2.5 (3.0) |
| Word Fluency Test | | |
| Letter | 26.5 (7.6)[b] | 31.7 (7.9) |
| Category | 35.4 (6.2)[a] | 48.4 (8.0) |

[a] $p < 0.01$.
[b] $p < 0.05$.
Standard deviations are in parenthesis.

ber of total responses. Regarding the classification of responses in the idea fluency test, the number of task-dependent responses was not significantly different between the two groups, but the number of task-modified and task-independent responses was significantly lower in the subjects with schizophrenia. In the design fluency test, there was no significant difference in the number of total responses between the groups. Regarding the classification of responses in the design fluency test, the number of task-modified responses was significantly lower but the number of task-dependent responses was significantly higher in the subjects with schizophrenia. The numbers of responses in the letter fluency test and category fluency test were significantly lower in the subjects with schizophrenia than in the healthy control group.

The subjects with schizophrenia demonstrated a significantly poorer performance on DST backward (patients, controls, 4.2 ± 0.8, 5.2 ± 1.6, respectively, $p = 0.007$) and RAVLT (11.0 ± 2.2, 12.8 ± 2.0, respectively, $p = 0.005$). The mean score of the MMSE for the schizophrenia group was 29.2 and did not differ significantly from the score of the healthy control group (29.5, $p = 0.280$). The LCT (108.8 ± 5.3, 111.0 ± 5.8, respectively, $p = 0.148$), DST forward (6.5 ± 0.9, 6.7 ± 1.0, respectively, $p = 0.661$), and WCST (categories achieved 5.0 ± 1.2, 5.0 ± 1.2, $p = 1.000$, perseverative errors 2.2 ± 2.1, 1.7 ± 2.1, $p = 0.428$, respectively) scores of the subjects with schizophrenia also did not significantly differ from those of subjects in the healthy control group.

### 3.2. *Associations among fluency test scores and between fluency test scores and other neuropsychological/demographic variables*

For multiplicity of the analysis, we did not employ any statistical adjustment for multiple comparisons, but more rigorous criteria for type I error, $P < 0.01$, was applied. In the idea and design fluency tests, only three kinds of sub-scores were analyzed.

In the correlations among fluency tests, the number of task-independent responses in the idea fluency test was significantly correlated with the number of task-independent responses in the design fluency test in the healthy control group ($r = 0.519$, $p = 0.007$). The CFT scores were not correlated with any scores of idea and design fluency tests in either group.

Any sub-scores of the idea and design fluency test did not significantly correlate with other neuropsychological variables in either group, except significant correlation between the number of task-independent responses in the design fluency test with RAVLT scores in the healthy control group ($r = 0.506$, $p = 0.008$).

None of the idea or design fluency test scores were significantly correlated with age, education period, disease duration, onset of disease, dosage of neuroleptics, or DIEPSS score.

## 4. Discussion

Although attention and executive functioning were relatively preserved in the subjects with schizophrenia, they demonstrated significant deficits in divergent thinking. The subjects with schizophrenia generated significantly fewer non-conventional and "high-quality" responses, such as task-modified and task-independent responses, than subjects in the healthy control group. But the ability to produce conventional responses, that is, task-dependent responses, was preserved in the subjects with schizophrenia. These findings suggest that patients with schizophrenia may have deficits in divergent thinking, with particular difficulty in generating responses requiring concept flexibility, conversion of viewpoint, originality, or novelty. Compared with our previous study [50], the current study demonstrated that relatively young patients with schizophrenia retained the ability to generate stereotyped responses but did not preserve the ability to generate high-quality responses clearly. Patients with schizophrenia may have the impairment based on a sequential hierarchy of divergent thinking from an elementary level to a more complex level.

No significant difference in the total number of responses on the design fluency test was observed between the two groups. Phillips et al. [40] reported that young patients with early-onset schizophrenia had significantly impaired category fluency, compared to healthy subjects, but no impairment in letter or design fluency. The qualitative scoring systems in the current study may be more sensitive to the characteristic differences in design fluency of patients with schizophrenia.

Psychomotor slowness may reduce fluency production quantitatively. It has been reported that verbal fluency is predicted by psychomotor speed, but not by memory or executive function [47]. But the present results showed the importance of qualitative evaluation of fluency or divergent thinking in patients with schizophrenia.

Lezak [31] reported that the ability to regulate one's own behaviour could be demonstrated on a test of flexibility that requires the subject to shift a course of thought or action according to the demands of the situation. Conceptual inflexibility appeared in concrete or rigid approaches to understanding and problem solving [31]. The present results may demonstrate the inflexibility of abstract thinking to generate new concepts starting from ordinary stimulus or situations in patients with schizophrenia. These findings are consistent with clinical descriptions of schizophrenia that include deficits in the productivity of thoughts and execution [6,7,41].

Dollfus et al. [12] reported that fluency test scores were significantly impaired in patients with schizophrenia, compared to the healthy subjects, while scores on a modified WCST performance did not differ between groups. They regarded the fluency impairments as endophenotypic markers of schizophrenia. Camozzato and Chaves [8] reported the discriminative and diagnostic values of verbal fluency tests for identifying patients with schizophrenia, compared with the WCST and MMSE. The present results also revealed the specific impairment in fluency or divergent thinking in patients with schizophrenia, in contrast to performance of the WCST. Both fluency tests and the WCST evaluate cognitive flexibility or executive function [31], but the WCST requires the subject to identify correct responses, rather than generate new concepts.

The task-independent responses on the idea and design fluency tests were significantly correlated in the healthy control group. The ability to produce extremely original and novel responses seems to be independent of the modality of the task. Task-independent responses may be produced by some kind of modality-independent neurocognitive function.

The idea and design fluency test scores did not correlate with most of the neuropsychological variables. Unexpectedly, neither the idea nor design fluency test scores were correlated with category fluency related to semantic organization in either group. These findings may reveal that divergent thinking ability is relatively independent of other neurocognitive functions. Furthermore, the results in the current study demonstrated that divergent thinking ability in patients with schizophrenia did not depend on illness duration, age of onset, medication dosage, or extrapyramidal symptoms. McCrae et al. [33] demonstrated that productivity in divergent thinking was maintained in subjects up until their early forties. The divergent thinking task scores in the current study were not influenced by age in contrast to our previous study [50] because all the subjects in this study were 42 years old or younger, with a mean age of about 30 years old.

Findings related to the neural specificity of fluency deficits have been contradictory [40]. Letter fluency is dependent upon left prefrontal cortex and left inferior parietal cortex functioning [4,16,34]. On the other hand, category fluency recruits both frontal and temporoparietal brain regions [16,36]. Design fluency is typically impaired by right frontal lesions [4,25]. Idea fluency is impaired by frontal lesions, but the laterality is unclear [43]. Further research is needed to explore the effects of brain lesions or dysfunctional neural systems on divergent thinking.

Previous invetigators have shown that fluency is related to spontaneous flexibility [9], an aspect of the dysexecutive syndrome known as initiation [29], willed action [14] or effortful and volitional retrieval from the lexicon [15]. Further studies examining the relationship between divergent thinking and social functioning in real-life situations or community settings are needed. Research on deficits in divergent thinking in patients with schizophrenia may contribute to the development of cognitive and behavioral rehabilitation programs.
